# Dental anxiety among dental, medical, and nursing students of two major universities in the central region of the Kingdom of Saudi Arabia: a cross-sectional study

**DOI:** 10.1186/s12903-019-0757-x

**Published:** 2019-04-15

**Authors:** Reham AL Jasser, Ghaida Almashaan, Haya Alwaalan, Najla Alkhzim, Abdulrhman Albougami

**Affiliations:** 1 Department of Periodontics and Community Dentistry, College of Dentistry, King Saud University, PO Box 60169, Riyadh, 11545 Saudi Arabia; 2 College of Dentistry, King Saud University, Riyadh, Saudi Arabia; 3 Department of Nursing, College of Applied Medical Sciences, Majmaah University, Almajmaah, 11952 Saudi Arabia

**Keywords:** Anxiety scale, Dental, Medical students, Dental students, Nursing students

## Abstract

**Background:**

Dental anxiety is one of the most common fears that can eventually lead to avoidance of dental care. Knowing how students will respond to dental treatment will aid in increasing their awareness of oral health and overcoming this fear. The present study measured the prevalence of dental anxiety among dental, medical, and nursing students in Riyadh city, Kingdom of Saudi Arabia.

**Methods:**

A cross-sectional study including undergraduate dental, medical, and nursing students at King Saud University and Almajmaa University was conducted to assess dental anxiety using the Modified Dental Anxiety Scale. Descriptive statistics including means, standard deviations, and percentages were calculated. Group comparisons were analyzed using t-tests and analysis of variance. Multiple group comparisons were conducted using Tukey’s post-hoc test. *P* < 0.05 was considered as the significance level.

**Results:**

Two hundred twenty-four participants completed the questionnaire. Medical students accounted for most of the responses (40.6%), followed by nursing (31.7%), and dental students (27.7%). There was no significant difference in dental anxiety with regard to gender. Dental students exhibited the least dental anxiety. A significant difference was found between students with good dental experiences compared with those who had bad dental experiences.

**Conclusion:**

Dental students had the lowest level of anxiety and anxiety levels were affected by previous dental visits. Increasing awareness and knowledge about oral health, regular dental education, and incorporating dental knowledge into university curriculums can aid in eliminating the fear of dentistry among health sciences students.

## Background

Despite advancements and new technologies used in dental treatment, dental anxiety is still considered one of the most common fears [1]. It can be defined as an exaggerated psychological apprehensive response of an individual to perceived dental treatment and can affect people of any gender, age, or social status [2, 3]. Dental anxiety could lead to avoidance of dental treatment and affect the patient both psychologically and physically by affecting their eating habits, decreasing their confidence in their teeth, and avoiding smiling [4]. This process could lead to deterioration in oral health and give rise to an unbearable problem after which the anxious individual will inevitably visit the dentist [5].

In previous research, dental anxiety was associated with traumatic past dental experiences, irregular dental visits, and a lack of dental awareness [2]. It can also affect the dentist-patient relationship, causing an increase in chair time, inaccurate diagnosis, and treatment setbacks [5–7]. Dental anxiety may affect the dentist as well, by increasing the dentist’s level of stress while providing dental treatment [5].

Several studies have investigated dental anxiety in university students; specifically, how dental students compare with students in other disciplines of study. Al-Omari et al. concluded that dental students had lower levels of dental anxiety than non-dental students, owing to their dental education and awareness of most clinical procedures [3]. Another study conducted in Malaysia also found that dental students had lower levels of dental anxiety than medical and pharmacy students, and for similar reasons [5]. Furthermore Storjord et al. [8] showed differences in the degree of dental anxiety among dental students according to their education level. Senior dental students had lower levels of dental anxiety than junior students, owing to more exposure to different dental procedures during their years spent in college, which was considered as a form of exposure therapy (environmental habituation). Thus, dental education along with clinical experience might reduce this anxiety [8]. All previously discussed studies highlighted the significance of lowering dental anxiety, which can lead to better acceptance of dental treatment, and thereby improve quality of life. This can be initiated by determining the level of dental anxiety initially present and its relationship to the amount of personal knowledge regarding dentistry.

Therefore, the purpose of the present study was to measure the prevalence of dental anxiety among dental, medical, and nursing students in Riyadh city, Kingdom of Saudi Arabia. The Modified Dental Anxiety Scale was used to assess dental anxiety. Additionally, we explored differences in the degree of dental anxiety according to gender, field of study, and dental treatment experience.

## Methods

### Study sample

The sample size was determined by G*Power software, with a confidence level set at 95%, power set at 80%, and a moderate effect size, which indicated a final sample size of 200 subjects. However, to avoid a low response rate, which could affect the sample size, a larger sample than that calculated was recruited, based on the assumption that not every individual in the sample would respond to the study. A final sample of 300 participants was targeted using purposive sampling.

Using a cross-sectional research design, the study involved survey research with dental, medical, and nursing undergraduate students from two different educational institutes in Riyadh, Saudi Arabia (King Saud University and Al Majmaa University). The students were in their third, fourth, and fifth year of college.

### Recruitment strategy

The researchers reserved a lecture room in which to conduct the study. Tables in the lecture room were marked with a reserved sign to increase the participants’ comfort level to sit and answer the survey questionnaires without interference or interruption by non-participants. Next, the researchers distributed flyers to recruit potential research participants. Interested participants could stop by the room to determine their eligibility. Eligible participants completed the informed consent forms prior to completing the survey questionnaires and returning them to the researcher. The objective of the study was explained to the students and any questions they had were answered. The anticipated time to complete the consent form and questionnaire was approximately 30 min. Finally, a total of 300 eligible students participated.

All responses were recorded anonymously and voluntarily. Students were informed that they have the right to discontinue from the study at any point in time without any consequence, and that the confidentiality of their responses would be maintained.

The Arabic version of the Modified Dental Anxiety Scale (MDAS) was used to assess dental anxiety [9]. The MDAS is considered a valid and reliable measure [5] and consists of five questions as follows: (1) If you went to your dentist for treatment tomorrow, how would you feel? (2) If you were sitting in the waiting room, how would you feel? (3) If you were about to have a tooth drilled, how would you feel? (4) If you were about to have your teeth scaled and polished, how would you feel? (5) If you were about to have a local anesthetic injection in your gum, how would you feel? Each question has five responses ranging from 1 (*not anxious*) to 5 (*extremely anxious*). The MDAS score is the sum of the five responses and can range from 5 to 25. A score of 19 or above is considered as dentally anxious, 12–18 mildly dentally anxious, and 5–11 as not anxious at all. Cronbach’s alpha for the MDAS was 0.86 in the present study, suggesting good internal consistency reliability. In addition to the MDAS, participants were asked about their gender, age, college specialty, and dental treatment experiences.

Descriptive statistics including means, standard deviations, and percentages were calculated. Group comparisons were analyzed using two-tailed Student’s t-tests as well as one-way analysis of variance. Multiple group comparisons were conducted using Tukey’s post-hoc test. *P* < 0.05 was considered as the significance level.

## Results

Out of 300 students, a total of 224 participants completed the questionnaire. Table 1 provides statistics on the participants’ age, gender, and field of study. The majority of respondents were female (75.9%) and were 18–22 years old (54.5%). Most of the participants were studying medicine (40.6%), followed by nursing (31.7%) then dentistry (27.7%). The mean MDAS score of the sample was 14, and 65.2% of the students were categorized as having a low level of dental anxiety. Only 18 participants were considered to have significant dental anxiety (Tables 2 and 3). Fifty-two percent reported that they visit the dentist only when having a dental problem, and 51% reported that their last dental visit was 6 months ago.

**Table 1:** Distribution of participants according to age, gender, and field of study

*Gender*	Number (*n*)	Percent (%)
Male	54	24.1
Female	170	75.9
*Age*		
18–22	122	54.5
23–27	81	36.2
More than 30	21	9.4
*Field of study*		
Medicine	91	40.6
Nursing	71	31.7
Dentistry	62	27.7
Total	224	100.0

**Table 2:** Distribution of MDAS scores among participants

Score	Frequency (*n*)	Percent (%)
5	26	11.6
6	25	11.2
7	20	8.9
8	19	8.5
9	20	8.9
10	21	9.4
11	15	6.7
12	8	3.6
13	9	4.0
14	13	5.8
15	11	4.9
16	9	4.0
17	5	2.2
18	5	2.2
19	6	2.7
20	2	0.9
21	3	2.3
22	3	1.3
24	2	0.9
25	2	0.9
Total	224	100.0

**Table 3:** Distribution of anxiety levels among participants

	Frequency (*n*)	Percent (%)
Low anxiety	146	65.2
Moderate anxiety	41	18.3
High anxiety	19	8.5
Severe anxiety	18	8.0
Total	224	100.0

When examining gender and dental anxiety, there was no significant difference in MDAS score between men (*M* = 9.63, *SD* = 3.728) and women (*M* = 10.98, *SD* = 5.009) (*P* = 0.069). On the other hand, when the field of study was compared, significant differences were found between dental students (*M* = 9.15, *SD* = 4.136) and nursing students (*M* = 12.55, *SD* = 4.972) (*P* = 0.000). There was also a significant difference between medical students (*M* = 10.21, *SD* = 4.634) and nursing students (*P* = 0.004). However, there was no significant difference in dental anxiety between medical students and dental students (*P* = 0.480) (Tables 4 and 5).

**Table 4:** MDAS mean scores of medical, dental, and nursing students

	Mean	Std. deviation
Medicine	10.21	4.634
Dentistry	9.15	4.136
Nursing	12.55	4.872

**Table 5:** Multiple comparisons among fields of study

(I) Field of Study	(J) Field of Study	Mean Difference (I-J)	Standard Error	*P* value	95% Lower Bound
Medicine	Nursing	−2.341	.725	.004	−4.09
	Dentistry	1.064	.754	.480	−.76
Nursing	Dentistry	3.404	.796	.000	1.48
	Medicine	2.341	.725	.004	.59
Dentistry	Nursing	−3.404	.796	.000	−5.33
	Medicine	−1.064	.754	.480	−2.88

When examining dental experiences, participants with good dental experiences had lower scores on the MDAS (*M* = 10.13, *SD* = 4.364) compared to those who had bad dental experiences (*M* = 15.45, *SD* = 5.621) (*P* = 0.000). Additionally, participants who had visited a recommended or previously known dentist had lower scores on the MDAS (*M* = 9.74, *SD* = 4.070) compared to those who were meeting a dentist for the first time (*M* = 14.62, *SD* = 5.504) (*P* = 0.000).

## Discussion

The purpose of the present study was to investigate the prevalence of dental anxiety among dental, medical, and nursing students in Riyadh city, Kingdom of Saudi Arabia. To this end, the Modified Dental Anxiety Scale was used to assess dental anxiety in the participants. Overall, the mean scores for the three categories of students indicate they were not dentally anxious at all or were mildly dentally anxious. Findings of this study revealed that dental students had a lower level of dental anxiety than medical students, although both groups’ scores indicate they were not dentally anxious at all. Nursing students had the highest score, which indicated they were mildly dentally anxious.

The results in the present study are in agreement with those reported by Al-Omari and Al-Omiri, in which dental students had lower levels of dental anxiety when compared to medical students [3]. Similarly, Thomas et al. showed that dental students had lower levels of dental anxiety compared to medical and nursing students [2]. These findings can be justified by the knowledge and background that dental students gain through their studies, which would aid in reducing their anxiety by knowing some of the details about each dental procedure. However, somewhat contrary to prior research, there was no statistically significant difference in dental anxiety between medical and nursing students, even though their scores fell in different clinical ranges [10]. Thomas et al. and Al-Omari and Al-Omiri found that medical students experienced high levels of dental anxiety compared to students from other non-health fields of study [2, 3]. It can be hypothesized that when comparing nursing and medical fields of study, the students have similar knowledge owing to sharing various courses, as well as sharing relatively similar exposure to the same professional environment, which may explain their different perception of dental interventions compared to dental students. Overall, and consistent with the previously mentioned studies, it can be argued that the knowledge gained through dental education is a major factor in reducing dental anxiety. This observation is supported by other studies that have been done in other countries. For example, in 2014 a study of undergraduate students at Al-Quds University in Palestine found that dental students were less anxious compared to medical and pharmacy students. Similarly, an Indian study revealed that non-dental students expressed more dental anxiety due to lack of dental education in their curriculum [2, 3, 10].

There were interesting findings when examining gender and past dental experiences in relation to dental anxiety. Male and female students did not differ in their level of dental anxiety, which is in agreement with another study that was conducted in the Kingdom of Saudi Arabia [11]. However, it is inconsistent with previous studies done in the same region that found that female students had higher levels of dental anxiety [2, 3, 12]. Not surprisingly, students who have had a bad dental experience reported higher levels of dental anxiety than those who have had good dental experiences. Thus, it is safe to say that positive experiences can potentially reduce dental anxiety levels.

Limitations of the present study include a larger number of females compared to males in the sample, which was due to limited access to male students within both institutes. Data were only collected from two institutes in the region, which may not be representative of the population because students’ knowledge and backgrounds may differ between different educational institutes in the country because of different curriculums. Therefore, further long-term studies with a larger sample size representing all institutes and regions in the country are recommended to more precisely assess the level of dental anxiety, so that suitable curriculums can be implemented to overcome this concern.

## Conclusion

Dental anxiety was lowest among dental students when compared to medical students and nursing students. In addition, the level of anxiety was influenced by whether previous dental experiences were good or bad. Therefore, eliminating the fear of dentistry could be achieved by providing regular dental education and implementing initiatives that promote awareness and knowledge about oral health [13]. As such, university curriculums should focus on increasing dental knowledge as a means of overcoming fear of dental treatment among university health sciences students [2].

## Abbreviation

MDAS: Modified dental anxiety scale

## References

[1] do Nascimento DL, da Silva Araújo AC, Gusmão ES (2011). Anxiety and fear of dental treatment among users of public health services. Oral Health Prev Dent.

[2] Thomas M, Kumar V, Sooraparaju S (2016). Dental anxiety among dental, medical, and nursing students in India and its correlation with their field of study. J Int Oral Health..

[3] Al-Omari WM, Al-Omiri MK (2009). Dental anxiety among university students and its correlation with their field of study. J Appl Oral Sci.

[4] Moore R, Brødsgaard I, Rosenberg N (2004). The contribution of embarrassment to phobic dental anxiety: a qualitative research study. BMC Psychiatry.

[5] Gunjal S, Pateel DGS, Parkar S (2017). Dental anxiety among medical and paramedical undergraduate students of Malaysia. Int J Dent..

[6] Armfield JM, Slade GD, Spencer AJ (2009). Dental fear and adult oral health in Australia. Community Dent Oral Epidemiol.

[7] Frazer M, Hampson S (1988). Some personality factors related to dental anxiety and fear of pain. Br Dent J.

[8] Storjord HP, Teodorsen MM, Bergdahl J (2014). Dental anxiety: a comparison of students of dentistry, biology, and psychology. J Multidiscip Healthc.

[9] Al-Nasser L, Yunus F, Ahmed A (2016). Validation of Arabic version of the modified dental anxiety scale and assessment of cut-off points for high dental anxiety in a Saudi population. J Int Oral Health.

[10] Abu Hantash RO, Abu Younis MH, Aker MM (2014). Dental anxiety and fear among medical field students at Al Quds University. Br J Med Res.

[11] Alshammary M, Alhumaid M, Alnejeem G, et al. Dental anxiety among medical field students in University of Hail, Saudi Arabia [internet]. Saspjournals.com; 2017. https://pdfs.semanticscholar.org/41f3/a2db7c25109d9282d48bab44d547103f8562.pdf. Accessed 11 Nov 2018.

[12] Sghaireen MG, Zwiri AM, Alzoubi IA (2013). Anxiety due to dental treatment and procedures among university students and its correlation with their gender and field of study. Int J Dent.

[13] Ogwo C (2018). Prevalence of dental anxiety among Western Illinois University students.

